# Literary Notes

**Published:** 1904-08

**Authors:** 


### LITERARY NOTES.

"Twenty-one years of post-graduate medical instruction" is the title of a handsome brochure containing a report of addresses at a dinner given in honor of Dr. D. B. St. John Roosa, at Delmonico's, New York, Tuesday evening, March 1, 1904. It is embellished with portraits of Dr. Roosa, Dr. A. W. Calhoun, Dr. William Osler, Dr. Clarence J. Blake, Dr. William J. Mayo, Dr. W. W. Keen, Hon. William Potter, Dr. William M. Polk, and Dr. Andrew H. Smith, all of whom delivered addresses. A loving cup, which is illustrated, was presented to Dr. Roosa by the corporation and faculty of the New York Post-Graduate Medical School and Hospital. A list of the guests, numbering 350, many from distant cities, is also published, the whole constituting a valuable souvenir of an event which the guest of honor may justly feel a pride in, and which also commemorates the twenty-one years since the inauguration of post-graduate medical instruction in this country.

---

The *Western Medical Review* has changed its form from double column quarto to standard octavo. This is commendable, as more befitting a monthly magazine, the weeklies only appearing to good advantage in the first named style. If the *Review* will now add to its improvement by placing its editorial material nearer the end instead of at the beginning of its pages, it will be a model monthly medical magazine.

---

Messrs. P. Blakiston's Son & Company made an interesting exhibit of medical books at the Atlantic City meeting of the American Medical Association last June. The reputation of this publishing house maintained through a long period of years, was never greater than at present. Among the new publications just ready, this house announces the following: A manual of surgical diagnosis, by James Berry, B.S., F.R.C.S., surgeon to the Royal Free Hospital, London, price $2.00; Case teaching in surgery, by Herbert L. Burrell, M.D., professor of clinical surgery, and John Bapst Blake, M.D., instructor in surgery, Harvard Medical School, price 75 cents; The treatment of some acute visceral inflammations, by D. B. Lees, M.A., M.D., physician to the Hospital for Sick Children and to Saint Mary's Hospital, London, price $1.50.

